# A microarray data analysis investigating the pathogenesis and potential biomarkers of autophagy and ferroptosis in intervertebral disc degeneration

**DOI:** 10.3389/fgene.2022.1090467

**Published:** 2023-01-04

**Authors:** Wenhao Kuang, Cong Jiang, Cheng Yu, Jinwei Hu, Yang Duan, Zhong Chen

**Affiliations:** Department of Spinal Surgery, Zhujiang Hospital, Southern Medical University, Guangzhou, China

**Keywords:** autophagy, DEGs, ferroptosis, hub genes, integrated bioinformatics, intervertebral disc degeneration

## Abstract

**Background:** Intervertebral disc degeneration (IDD) entails complex pathological changes and causes lower back pain (LBP). However, there is still a lack of understanding of the mechanisms involved in IDD, particularly regarding the roles of autophagy and ferroptosis. The current study used microarray data to investigate the pathogenesis of IDD and potential biomarkers related to autophagy and ferroptosis in IDD.

**Methods:** Differentially expressed genes (DEGs) were identified by analyzing the mRNA and miRNA expression profiles of IDD patients from the Gene Expression Omnibus (GEO). The protein-protein interaction network, Gene Ontology (GO) enrichment, Kyoto Encyclopedia of Genes and Genomes (KEGG) analysis, and gene set enrichment analysis (GSEA) were utilized. The Human Autophagy Database (HADb) and Ferroptosis Database were used in conjunction with hub genes to identify autophagy- and ferroptosis-related genes. The Transcription Factor -hub gene-miRNA network was constructed. Lastly, the expression of DEGs in normal and degenerated nucleus pulposus cells (NPCs) was investigated *via* the quantitative reverse transcription polymerase chain reaction (qRT-PCR).

**Results:** A total of 362 DEGs associated with IDD were identified. GO and KEGG analyses indicated that oxidative stress, extracellular matrix, PI3K-AKT signaling pathway, and ferroptosis were key factors in IDD occurrence. GSEA indicated that IDD was associated with changes in autophagy, iron ion homeostasis, extracellular matrix, and oxidative stress. Eighty-nine hub genes were obtained, including five that were autophagy-related and three that were ferroptosis-related. Of these, TP53 and SESN2 were the intersections of autophagy- and ferroptosis-related genes. In qRT-PCR analysis, CANX, SLC38A1, and TP53 were downregulated in degenerative NPCs, whereas GNAI3, SESN2, and VAMP3 were upregulated.

**Conclusion:** The current study revealed aspects of autophagy- and ferroptosis-related genes involved in IDD pathogenesis, warranting further investigation.

## Introduction

Lower back pain (LBP) affects people of all cultures and ages. It reportedly affects 700 million patients worldwide physically, psychologically, and economically and reduces the productivity of society by causing massive economic losses (Francisco et al., 2022). Intervertebral disc degeneration (IDD) is thought to be the most important component of LBP (Zhao et al., 2021). Intervertebral discs (IVDs) are composed of fibrous tissue and cartilage and have an avascular structure that contains three layers: the annulus fibrosus with its outer and inner parts, the central nucleus pulposus, and the endplate. The normal function of an IVD depends on the cells and stroma (Kos et al., 2019). Multiple studies have indicated that various pathological factors, such as extracellular matrix (ECM) degradation, oxidative stress, inflammatory responses, mitochondrial dysfunction, nutritional deficiency, abnormal mechanical loading, and epigenetic changes, are involved in IDD progression (Zhang G. Z. et al., 2020; Zhang et al., 2021; Liang et al., 2022). However, the regulatory mechanisms involved in IDD are complex; thus, many of them remain to be elucidated.

Autophagy is a process in which cells self-digest their own components. It nourishes cells during fasting, clearing them of excess or damaged organelles, misfolded proteins, and invading microorganisms (Levine and Kroemer 2008). In recent years, numerous studies have indicated that disordered autophagy may lead to IDD (Gong and Zhang 2021; Madhu et al., 2021). Studies investigating autophagy in IDD are still at a very early stage, and the physiological role of autophagy in normal IVDs and specific mechanisms involved in autophagy regulation in IVD cells remain unclear (Kritschil et al., 2022). More studies on relevant functions and mechanisms are needed to clarify the role of autophagy in IDD.

The term ferroptosis, coined in 2012 (Dixon et al., 2012), describes a unique form of cell death. It is driven by iron-dependent phospholipid peroxidation and regulated by multiple cellular metabolic events, such as redox homeostasis, mitochondrial activity, and metabolism of amino acids, lipids, and sugars, as well as many disease-related signaling pathways. Ferroptosis may be involved in many organ damage and degenerative pathologies (Jiang et al., 2021). Previous studies have suggested that ferroptosis might play a role in IDD (Zhang GZ. et al., 2020; Lu et al., 2021).

The current study used microarray data analysis to investigate IDD pathogenesis and potential biomarkers related to autophagy or ferroptosis in IDD.

## Materials and methods

### Microarray datasets

Four datasets were retrieved from the Gene Expression Omnibus (GEO) database (Edgar et al., 2002; Barrett et al., 2012) (http://www.ncbi.nlm.nih.gov/geo): two mRNA expression datasets [GSE23130 (Gruber et al., 2012) and GSE15227 (Gruber et al., 2009)] and two miRNA expression datasets [GSE63492 (Liu et al., 2015) and GSE116726 (Ji et al., 2018)]. GSE15227 contained 12 healthy samples and three IDD samples, GSE23130 contained 15 healthy samples and eight IDD samples, GSE116726 contained three healthy samples and three IDD samples, and GSE63492 contained five healthy samples and five IDD samples.

### Identification of differentially expressed genes and hub genes

The GEO2R network tool (https://www.ncbi.nlm.nih.gov/geo/geo2r/) is based on the R software “LIMMA” and GEO query (Barrett et al., 2012), and it was used in the current study to identify differentially expressed mRNAs and miRNAs in healthy and IDD samples. A log2 fold change of >1 and *p* of <.05 were used as the criteria to identify differentially expressed genes (DEGs). The intersections of the DEGs in the two mRNA datasets and two miRNA datasets were used to obtain differentially expressed mRNAs and miRNAs. Target genes were predicted from the differentially expressed miRNAs using MIRNET (https://www.mirnet.ca/), and hub genes were identified after crossing the target genes with the differentially expressed mRNAs. VEEN diagrams were then constructed to detect and visualize the intersections of these databases.

### Gene set enrichment analysis

“c2.all.v7.4.symbols” and “c5.all.v7.4.symbols” gene sets in the MSigDB database (Liberzon et al., 2015) were used as reference gene sets. Then, gene set enrichment analysis (GSEA) was performed (Subramanian et al., 2005) on GSE23130 and GSE15227.

### Protein-protein interaction network construction

Genes with a minimum required interaction score of >.4 were selected to construct a differentially expressed mRNA-associated protein-protein interaction (PPI) network using the STRING database. The DEGs were functionally annotated using the Cytoscape 3.8.0 plug-in “clueGO” (Bindea et al., 2009).

### Functional enrichment analyses of hub genes

Gene ontology (GO) and Kyoto Encyclopedia of Genes and Genomes (KEGG) functional enrichment analyses of the hub genes were performed using the R software “clusterProfiler” (Yu et al., 2012), and the results were visualized with the R software “ggplot2”.

### Transcription factor-hub gene-miRNA network construction

Network Analyst (https://www.networkanalyst.ca/) was used to predict transcription factors that could regulate hub genes to construct the transcription factor-hub gene-miRNA network, and this network was visualized using Cytoscape (v.3.8.0).

### Autophagy-related and ferroptosis-related genes in IDD

Autophagy-related genes were obtained from the Human Autophagy Database (HADb) (http://www.autophagy.lu/index.html) and then intersected with the hub genes to obtain the autophagy-related genes in IDD. Similarly, driver, suppressor, and marker data were obtained from the Ferroptosis Database (FerrDb) (http://www.zhounan.org/ferrdb), with removed duplication, and then intersected to obtain ferroptosis-related genes for subsequent analysis.

### Isolation and culture of nucleus pulposus cells

Normal human nucleus pulposus tissue was collected, washed with phosphate-buffered saline (PBS), and then cut into pieces. After digestion with .2% collagenase type II (Thermo Fisher Scientific, United States) for 2 h, followed by .25% digestion with trypsin for .5 h, the samples were washed again with PBS. After centrifugation, they were transferred to culture flasks with Dulbecco’s Modified Eagle’s Medium)/F12 medium containing 10% fetal bovine serum (Thermo Fisher Science, United States) and 1% penicillin-streptomycin (Thermo Fisher Science, United States) at 37°C in a 5% CO_2_ incubator for cell isolation and cultivation. The cell culture medium was replaced on the fifth day and every two or three days thereafter. After 80%–90% confluency was reached, nucleus pulposus cells (NPCs) were passaged. Third-passage NPCs were used in subsequent experiments. The experimental protocol was approved by the Ethics Committee of Zhujiang Hospital of Southern Medical University.

### Quantitative reverse transcription-PCR

NPCs were divided into normal and degeneration groups. In the normal group, the cell culture medium was replaced every 48–72 h. In the degeneration group, the NPCs were treated with 100 ng/ml tumor necrosis factor (TNF)-α for 24 h. Total RNA was extracted and reverse-transcribed *via* a kit in accordance with the manufacturer’s instructions (Accurate Biotechnology, China). Quantitative reverse transcription polymerase chain reaction (qRT-PCR) was performed using the CFX Connection Real-Time System (Bio-Rad, United States). The 2^−ΔΔCt^ method was used to evaluate gene expression relative to GAPDH expression. Sequence fragments of RNAs are shown in Table 3.

### Statistical analysis

Data were expressed as mean ± the standard deviation. Gene expression levels of clinical samples were compared using the independent sample *t*-test. Benjamini–Hochberg procedure was used to reduce the false-positive rate of multiple tests. A *p* of <.05 was considered statistically significant. All statistical analyses were conducted using R programming (https://www.r-project.org/, version 4.0.2).

## Results

### Identification of DEGs and hub genes

**FIGURE 1:**
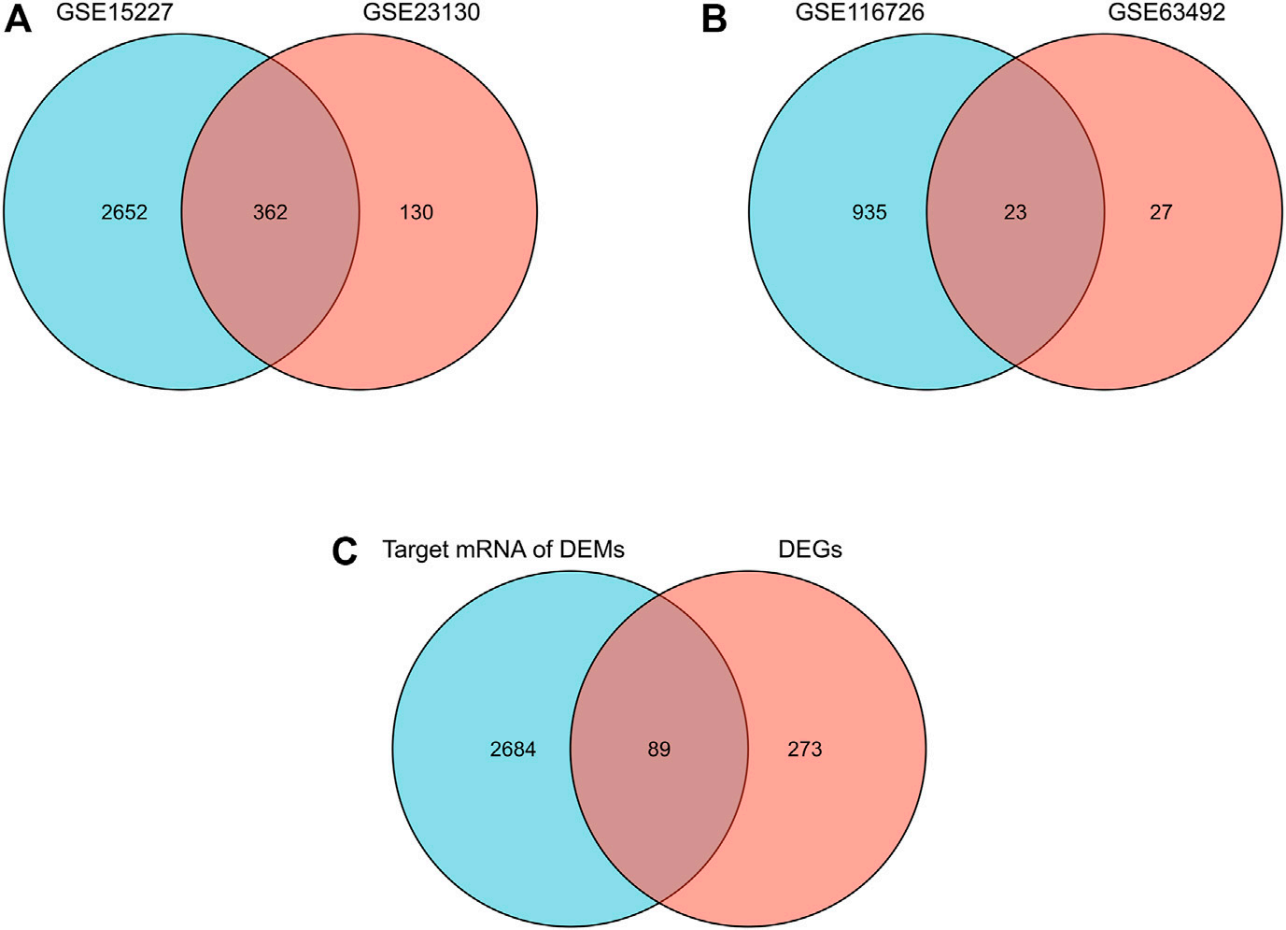
Differential expression of mRNAs, miRNAs, and hub genes in IDD. **(A)** Venn diagram of differentially expressed mRNAs in the two mRNA datasets. **(B)** Venn diagrams of differentially expressed miRNAs in two miRNA datasets. **(C)** Venn diagram of hub genes in IDD.

A total of 1,817 upregulated and 1,689 downregulated mRNA and 470 upregulated and 538 downregulated miRNA were identified. Of these, 362 overlapping mRNA were defined as differentially expressed mRNA, and 23 overlapping miRNAs were defined as differentially expressed miRNAs (Figures 1A,B). A total of 2773 target mRNAs were predicted from these 23. Eighty-nine hub genes were identified by intersecting the 2773 target mRNAs with 362 differentially expressed mRNAs (Figure 1C; Table 1).

**TABLE 1:** 89 hub genes.

ATP6AP1	CANX	CCND2	CDC42	COL1A1	EEF1A1	IGFBP5	LASP1
PRELP	PTMA	SET	TP53	EIF4H	MATR3	ZC3H11A	CELF1
BNC2	TMEM214	SLC38A1	GNAI3	MYO10	PPIC	FOXP1	FAM46A
THBS2	RGS5	HYPK	SERPING1	FUS	GM2A	NDUFC2	RPL12
TOR1AIP1	TNPO2	ANXA4	TRAM1	CMTM6	SETD3	CAPZA1	HLA-A
NAV1	PRKAR2A	TMED2	SULF2	APLP2	HMGN2	DAZAP2	ADAR
SGK1	VAMP3	SESN2	GPX1	ASPH	ZFP36L1	MAT2A	PDGFRA
TMEM248	COL1A2	GNB1	CRTAP	TMED10	SCRG1	ITGA11	GPBP1
ZCCHC24	HLA-B	PSMB5	RPL35	DYNLT1	CYP1B1	PPT1	SUMO2
NFE2L1	TRIM29	VOPP1	RPL27A	MSN	CLU	PGAM1	DHX40
FBXO28	GRB2	AMOTL2	NRP2	UGDH	TPM3	TGOLN2	SSR1
UBLCP1							

### PPI network analysis

**FIGURE 2:**
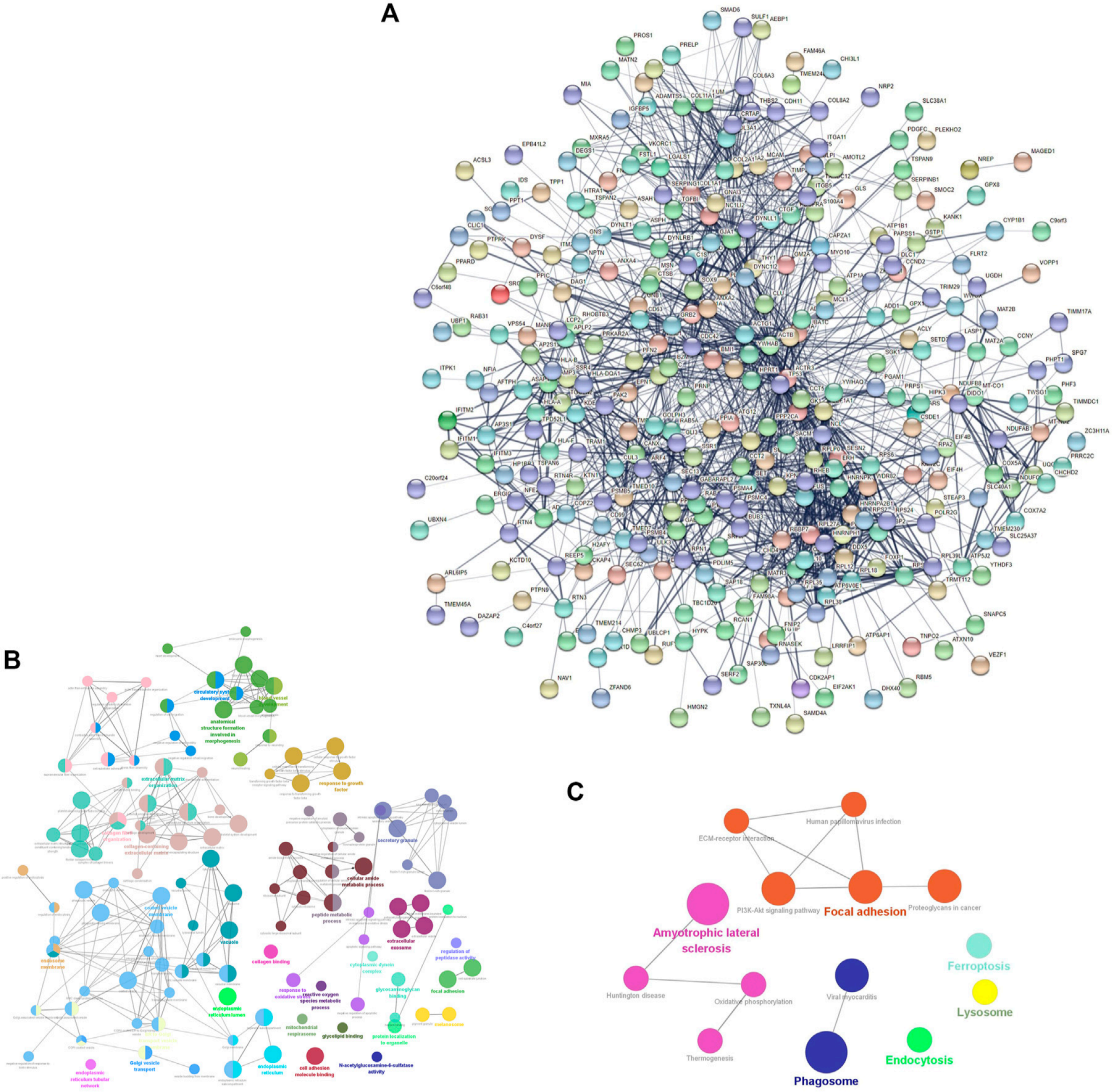
mRNA-related PPI networks in IDD. **(A)** PPI network of differentially expressed mRNAs in IDD. **(B)** clueGO-GO analysis of genes in PPIs that had differentially expressed mRNAs associated with IDD. **(C)** clueGO-KEGG analysis of genes in PPIs that had differentially expressed mRNAs associated with IDD.

The PPI network consisted of 361 nodes and 1595 edges (Figure 2A). The average node degree was 8.84, and the local clustering coefficient was .397. The three proteins with the highest node degrees were ACTB (90 nodal degrees), TP53 (69 nodal degrees), and FN1 (60 nodal degrees).

### GO and KEGG enrichment analyses of DEGs

GO and KEGG analyses of the DEGs obtained from the PPI network were performed to verify the functions of differentially expressed mRNAs in IDD. GO analysis results indicated changes in responses to oxidative stress, reactive oxygen species (ROS) metabolic processes, transforming growth factor beta (TGF-β) receptor signaling pathway, TGF-β, growth factor binding, collagen-containing ECM, ECM organization, mitochondrial respirasome, apoptotic signaling pathway, intrinsic apoptotic signaling pathway in response to oxidative stress, and lysosomes (Figure 2B). KEGG pathway enrichment analysis indicated that the DEGs were significantly enriched in ferroptosis, oxidative phosphorylation, PI3K-Akt signaling pathway, lysosomes, and ECM-receptor interaction (Figure 2C).

### GSEA

**FIGURE 3:**
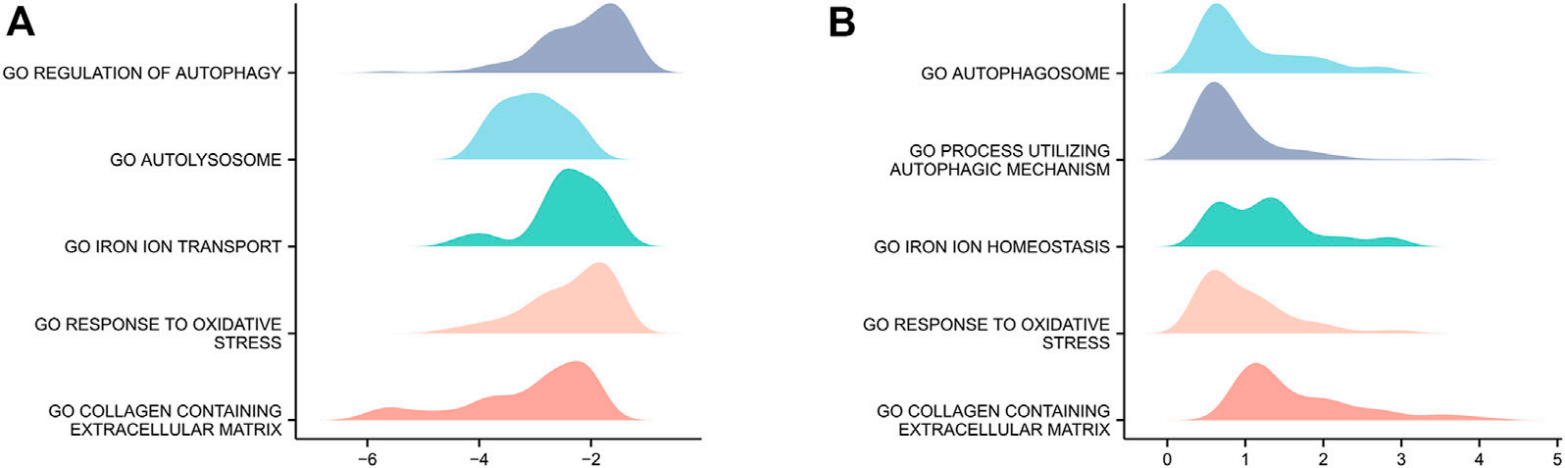
GSEA of the two datasets. **(A)** GSEA of genes in the GSE15227 dataset. **(B)** GSEA of genes in the GSE23130 dataset.

Genes in GSE15227 were significantly involved in five biological functions: regulation of autophagy, autolysosomes, iron ion transport, responses to oxidative stress, and collagen-containing ECM (Figure 3A). Genes in GSE23130 were also significantly involved in five biological functions: autophagosomes, processes utilizing autophagic mechanisms, iron ion homeostasis, responses to oxidative stress, and collagen-containing ECM (Figure 3B).

### GO and KEGG enrichment analyses of hub genes

**FIGURE 4:**
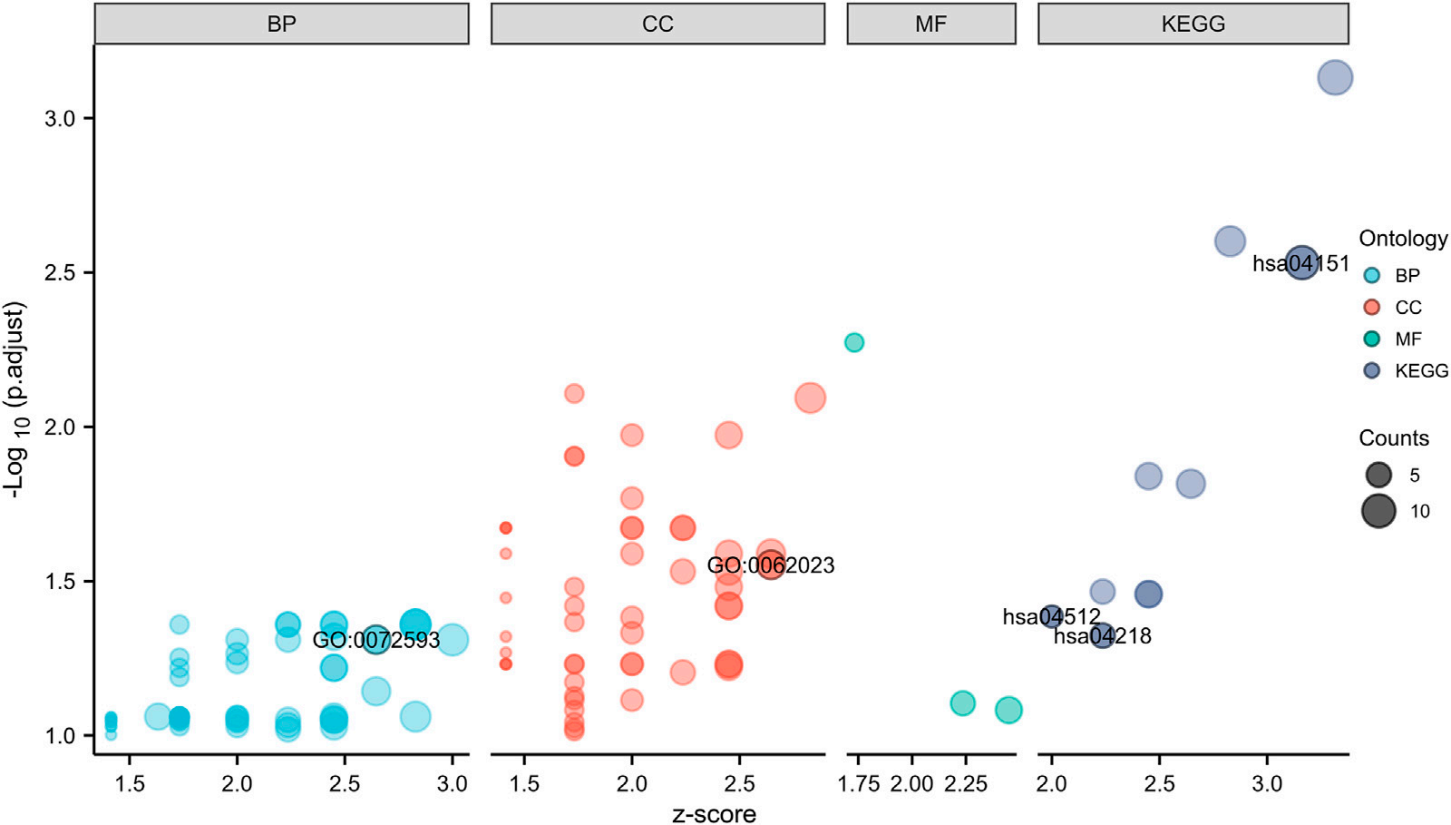
GO and KEGG enrichment analyses of hub genes.

In GO analysis, hub genes were mainly enriched in ROS metabolic processes and collagen-containing ECM. In KEGG pathway analysis, hub genes were mainly enriched in the PI3K-Akt signaling pathway, ECM-receptor interaction, and cellular senescence (Figure 4; Table 2).

**TABLE 2:** The GO and KEGG enrichment analyses of hub genes in IDD.

Ontology	ID	Description	Counts	*p*.Adjust
BP	GO:0070208	Protein heterotrimerization	3	.04370571
	GO:0002474	Antigen processing and presentation of peptide antigen *via* MHC class I	5	.04370571
	GO:0007596	Blood coagulation	8	.04370571
	GO:0006613	Cotranslational protein targeting to membrane	5	.04370571
	GO:0002478	Antigen processing and presentation of exogenous peptide antigen	6	.04370571
	GO:0007599	Hemostasis	8	.04370571
	GO:0050817	Coagulation	8	.04370571
	GO:0019884	Antigen processing and presentation of exogenous antigen	6	.04388518
	GO:0048002	Antigen processing and presentation of peptide antigen	6	.04788005
	GO:0071230	Cellular response to amino acid stimulus	4	.04894962
	GO:0051291	Protein heterooligomerization	5	.04894962
	GO:0072593	Reactive oxygen species metabolic process	7	.04894962
	GO:0034248	Regulation of cellular amide metabolic process	9	.04894962
CC	GO:0042588	Zymogen granule	3	.0078053
	GO:0005788	Endoplasmic reticulum lumen	8	.00806168
	GO:0030662	Coated vesicle membrane	6	.01064114
	GO:0012507	ER to Golgi transport vesicle membrane	4	.01064114
	GO:0071556	Integral component of lumenal side of endoplasmic reticulum membrane	3	.01246273
	GO:0098553	Lumenal side of endoplasmic reticulum membrane	3	.01246273
	GO:0055038	Recycling endosome membrane	4	.01704253
	GO:0031091	Platelet alpha granule	4	.02127679
	GO:0030134	COPII-coated ER to Golgi transport vesicle	4	.02127679
	GO:0005583	Fibrillar collagen trimer	2	.02127679
	GO:0042589	Zymogen granule membrane	2	.02127679
	GO:0098643	Banded collagen fibril	2	.02127679
	GO:0055037	Recycling endosome	5	.02127679
	GO:0005798	Golgi-associated vesicle	5	.02127679
	GO:0030660	Golgi-associated vesicle membrane	4	.0257672
	GO:0030133	Transport vesicle	7	.0257672
	GO:0030135	Coated vesicle	6	.0257672
	GO:0031588	Nucleotide-activated protein kinase complex	2	.0257672
	GO:0062023	Collagen-containing extracellular matrix	7	.02799188
	GO:0030658	Transport vesicle membrane	5	.02942768
	GO:0005938	Cell cortex	6	.02942768
	GO:0022625	Cytosolic large ribosomal subunit	3	.03300919
	GO:0034774	Secretory granule lumen	6	.03300919
	GO:0098644	Complex of collagen trimers	2	.03581022
	GO:0060205	Cytoplasmic vesicle lumen	6	.03803232
	GO:0031983	Vesicle lumen	6	.03803232
	GO:0033116	Endoplasmic reticulum-Golgi intermediate compartment membrane	3	.03803232
	GO:0030176	Integral component of endoplasmic reticulum membrane	4	.04144671
	GO:0030670	Phagocytic vesicle membrane	3	.04288623
	GO:0031227	Intrinsic component of endoplasmic reticulum membrane	4	.04649678
	GO:0042611	MHC protein complex	2	.0478303
MF	GO:0048407	Platelet-derived growth factor binding	3	.00533266
KEGG	hsa05165	Human papillomavirus infection	11	.00073907
	hsa04510	Focal adhesion	8	.00250796
	hsa04151	PI3K-Akt signaling pathway	10	.00294227
	hsa04145	Phagosome	6	.01445851
	hsa05163	Human cytomegalovirus infection	7	.0152956
	hsa04926	Relaxin signaling pathway	5	.03419516
	hsa05203	Viral carcinogenesis	6	.03483872
	hsa05205	Proteoglycans in cancer	6	.03483872
	hsa04512	ECM-receptor interaction	4	.04114587
	hsa04218	Cellular senescence	5	.04747178

### TF-hub Gene-miRNA network

**FIGURE 5:**
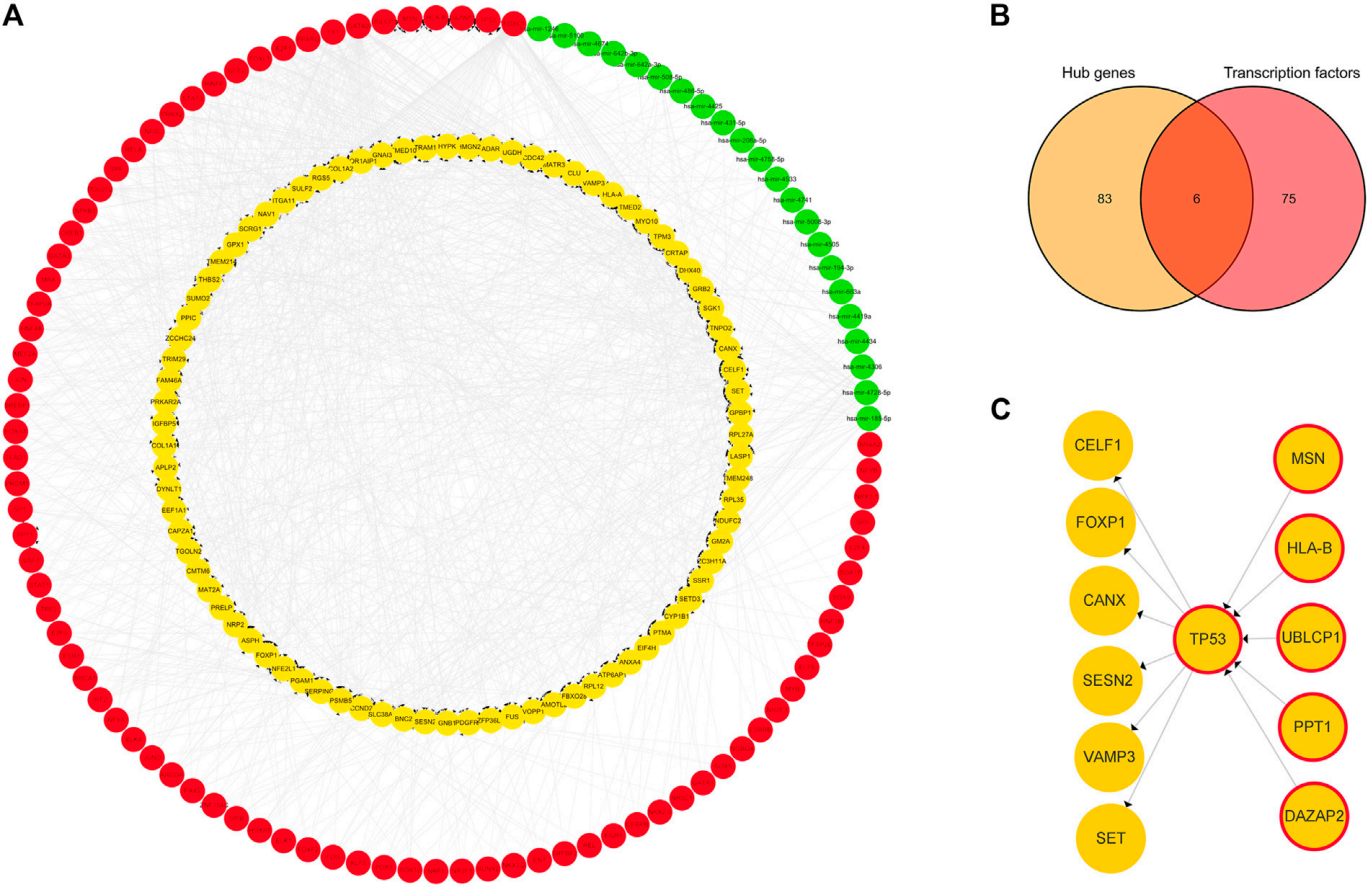
TF-hub gene-miRNA network. **(A)** Red nodes represent TFs, green nodes represent miRNAs, and yellow nodes represent hub genes. **(B)** Venn diagram of hub genes acting as TFs in IDD. **(C)** Yellow nodes represent hub genes, and yellow nodes with red borders represent hub genes acting as transcription factors.

A TF-hub gene-miRNA network containing 89 mRNAs, 22 miRNAs, and 81 TFs was generated. The top targeted miRNA hub gene was TP53, which was modulated by seven miRNAs. The top targeted TF hub gene was GPBP1, which was modulated by 20 TFs. The TF that regulated the most hub genes was FOXC1, which regulated 56 hub genes. The DEM that regulated the most hub genes, 22, was hsa-mir-185-5p (Figure 5A). Six genes were obtained after the 81 predicted TFs were intersected with the 89 hub genes: TP53, DAZAP2, HLA-B, MSN, UBLCP1, and PPT1. This indicated that they are simultaneously regulated by TFs and differentially expressed miRNAs and also regulate some hub genes (Figure 5B). TP53 was evidently a key juncture of the network (Figure 5C).

### Autophagy-related and ferroptosis-related hub genes in IDD

**FIGURE 6:**
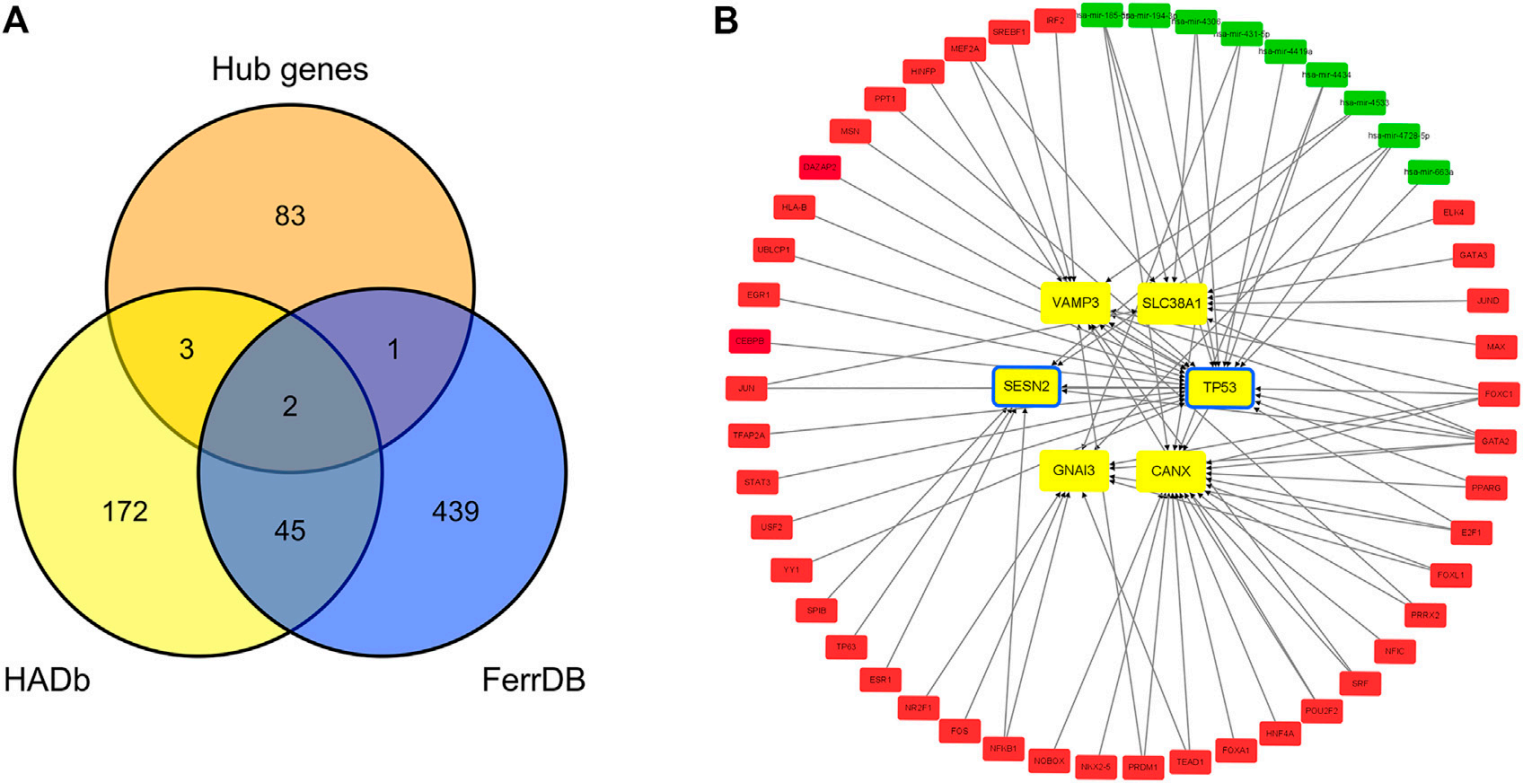
Autophagy-related and ferroptosis-related hub genes in IDD and associated TF-hub gene-miRNA network. **(A)** Autophagy- and ferroptosis-related hub genes in IDD, indicating that there are two genes simultaneously associated with both autophagy and ferroptosis. **(B)** Red rectangles represent TFs, green rectangles represent miRNAs, yellow and blue rectangles represent autophagy and ferroptosis genes, respectively, and yellow rectangles with a blue frame represent genes associated with both autophagy and ferroptosis.

Two hundred and twenty-two autophagy-related genes and 487 ferroptosis-related genes were obtained. Intersecting 222 autophagy-related genes with 89 hub genes resulted in the identification of five autophagy-related hub genes in IDD: CANX, GNAI3, SESN2, TP53, and VAMP3. Intersecting 487 ferroptosis-related genes with 89 hub genes resulted in the identification of three ferroptosis-related hub genes in IDD: TP53, SLC38A1, and SESN2. TP53 and SESN2 were simultaneously associated with both autophagy and ferroptosis (Figure 6A). The related TF-hub gene-miRNA network was then constructed (Figure 6B).

### Expression levels of hub genes

**FIGURE 7:**
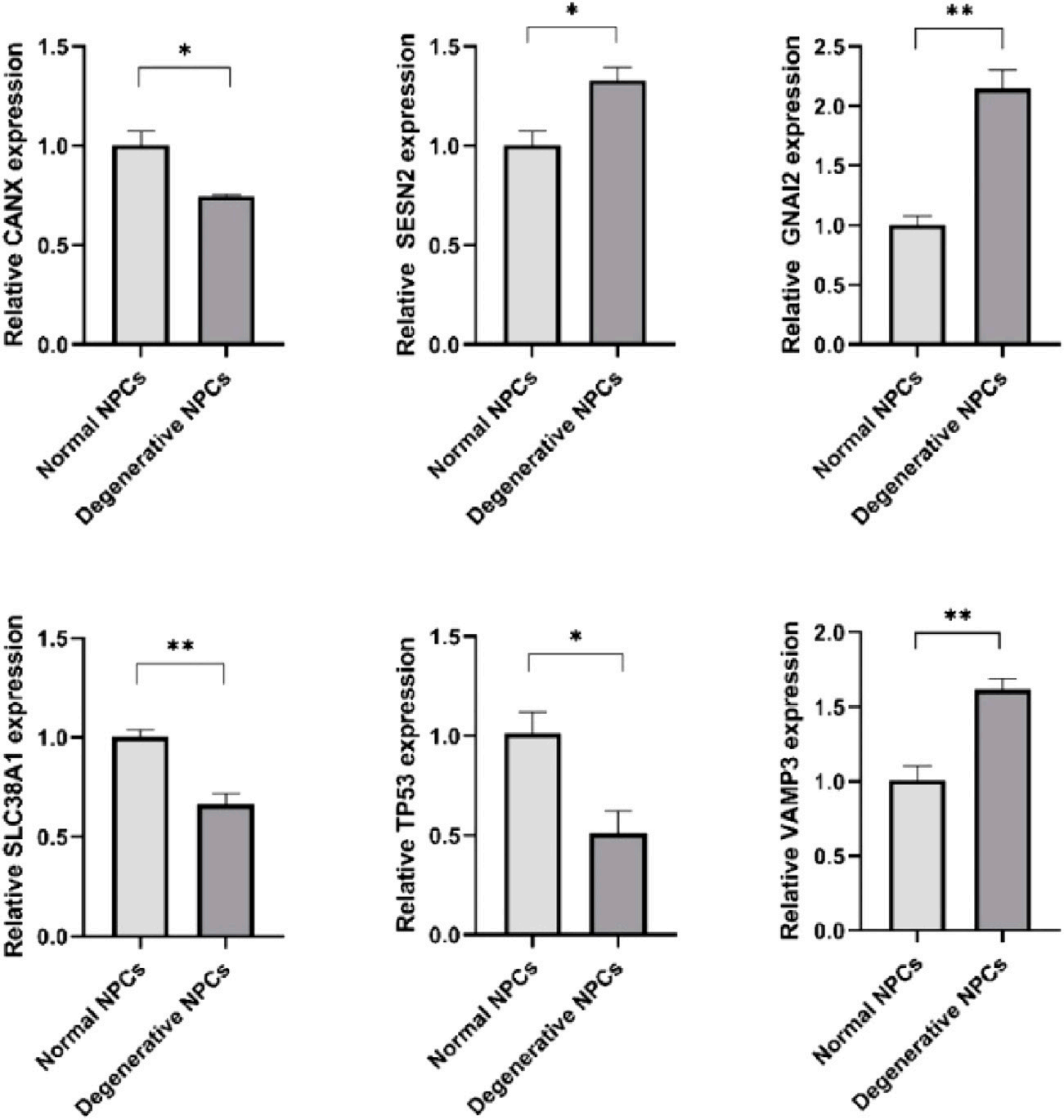
Expression levels of hub genes in normal NPCs and degenerated NPCs. *p < 0.05; **p < 0.01; ***p < 0.001; ns, not significant.

CANX, SLC38A1, and TP53 were downregulated in degenerative NPCs compared to normal NPCs, whereas GNAI3, SESN2, and VAMP3 were upregulated (Figure 7) (Table 3).

**TABLE 3:** Primer sequences.

Gene	Forward primer	Reverse primer
CANX	TAC​CAG​CCT​CAG​CCT​CCC​AAA​G	GCC​AAG​ATC​ACG​CCA​CTA​CAC​TC
GNAI3	TTG​GCA​CTG​GCA​TCC​TTG​TCT​TAT​C	TGC​TCC​TCC​CTC​AGT​CTT​CTC​ATT​C
SESN2	GAG​GCA​GGA​GAA​TCG​CTT​GAA​CC	TTG​AGA​CGG​AGT​ATC​GCT​CTT​GTT​G
SLC38A1	GGC​ACC​ACA​GGG​AAG​TTC​GTA​ATC	ACG​ATG​AAG​AGG​TAG​CTC​AGC​ATT​G
TP53	AGG​ACA​AGA​AGC​GGT​GGA​GGA​G	TGT​TGT​TGG​GCA​GTG​CTA​GGA​AAG
VAMP3	GAG​GCA​GGA​GAA​TGG​CAT​GAA​CC	ACG​GAG​ACT​TGC​TCT​GTC​ACC​TAG
GADPH	GCA​CCG​TCA​AGG​CTG​AGA​AC	TGG​TGA​AGA​CGC​CAG​TGG​A

## Discussion

IDD is an important cause of LBP and involves complex pathophysiological changes and molecular mechanisms. Research on the occurrence and developmental mechanisms involved in IDD has been limited, particularly regarding mechanisms relating to autophagy and ferroptosis. In the current study, microarray and bioinformatics analyses were used to investigate possible molecular mechanisms involved in IDD occurrence and development, and potential biomarkers were predicted.

Both GO and KEGG enrichment analyses of DEGs and hub genes highlighted the importance of oxidative stress, ECM, and PI3K-AKT signaling pathway, and all three of these were related to autophagy and ferroptosis. Activation of the PI3K-AKT signaling pathway can increase ECM content, prevent apoptosis, promote cell proliferation, induce or prevent autophagy, mitigate oxidative damage, and contribute to adaptation to a hypoxic microenvironment, thereby preventing IDD (Ouyang et al., 2017). Exosomes of the cartilage endplate activate the PI3K/AKT/autophagy pathway, inhibit NPC apoptosis, and slow the IDD process (Luo et al., 2021). Islet amyloid polypeptide regulates gene expression responsible for ECM anabolism and catabolism and controls the interaction between apoptosis and NPC autophagy *via* the PI3K/AKT/mTOR and p38/JNK MAPK signaling pathways (Wu et al., 2017). XIST may act as a competitive endogenous RNA of MIRI-19, and XIST/MIRI-19/PTEN crosstalk reduces NPC viability, promotes NPC degeneration, and induces NPC autophagy in IVD tissues *via* the PI3K/Akt signaling pathway inactivation, slowing IDD development (Chen W et al., 2021). Bone marrow mesenchymal stem cell-derived extracellular vesicles harboring circ_0072464 inhibit NPC ferroptosis by inhibiting miR-431 and upregulating NRF2, which promotes NPC matrix synthesis and proliferation, alleviating IDD (Yu et al., 2022). Under oxidative stress, ferroprotein imbalance leads to NP intercellular iron overload, resulting in NPC ferroptosis and accelerating IDD progression *in vivo* (Lu et al., 2021). Homocysteine promotes IDD development by upregulating oxidative stress and ferroptosis in the nucleus pulposus by enhancing GPX4 methylation (Zhang G. Z. et al., 2020). Previous studies have indicated that oxidative stress, ECM, and PI3K-Akt signaling pathway might be clinical biomarkers or therapeutic indicators in IDD, which is consistent with the current study. Based on the present analysis and previous studies, we suggest that the roles of autophagy and ferroptosis in IDD warrant attention.

TP53 encodes a tumor suppressor protein containing transcriptional activation, DNA binding, and oligomerization domains. The p53-mediated transcriptional suppression of SLC7A11 promotes ferroptosis in cancer cells. p53 R273H and R175H inhibit the expression of SLC7A11 by not binding to DNA and by inhibiting the activity of other transcription factors, suggesting that the expression of this core ferroptosis regulator is regulated by an integrated network of transcription factors (Chen X et al., 2021). p53 can enhance ferroptosis by enhancing the expression of SAT1 (spermidine/spermine N1-acetyltransferase 1) and GLS2 (glutaminase 2) or by inhibiting that of SLC7A11 (solute carrier family seven member 11). On the other hand, p53 suppresses ferroptosis through the direct inhibition of DPP4 (dipeptidyl peptidase 4) activity or by the promotion of CDKN1A/p21 (cyclin-dependent kinase inhibitor 1A) expression (Kang et al., 2019). The loss of TP53 prevents nuclear accumulation of DPP4, which promotes plasma membrane-associated DPP4-dependent lipid peroxidation, leading to ferroptosis (Xie et al., 2017). Previous studies have demonstrated the central role of TP53 in the ferroptosis regulatory network, controlling ferroptosis by directly regulating the expression of downstream ferroptosis-related target genes or regulating the expression of other transcription factors to control the expression of death-related genes. Our results indicated that TP53 might regulate downstream ferroptosis-related target gene SESN2 to regulate ferroptosis, while TP53 might be regulated by other transcription factors (such as DAZAP2, HLA-B, MSN, UBLCP1, and PPT1) to form a complex and integrated regulatory network of transcription factors, which was consistent with previous studies.

SESN2 encodes a member of the sestrin family of PA26-related proteins. SESN2 can play an antioxidant role in sepsis, downregulating the ATF4-CHOP-CHAC1 signaling pathway and inhibiting ferroptosis in dendritic cells (Li et al., 2021). Ferroptosis-mediated SESN2 induction protects against iron overload and ferroptosis-induced liver injury, relying on NRF2 (Park et al., 2019). Based on existing studies, we believe that TP53 may be a central gene in the regulatory network of ferroptosis in IDD, and it may play an important role in IDD by regulating SESN2 or a complex transcription factor regulatory network to control ferroptosis.

SLC38A1 functions as a sodium-dependent amino acid transporter. As a competitive endogenous RNA, long non-coding RNA ZFAS1 promotes the conversion of lung fibroblasts to myofibroblasts and ferroptosis *via* the MIR-150-5P/SLC38A1 axis (Yang et al., 2020). OGFRP1 regulates lung cancer cell proliferation and ferroptosis by inhibiting miR-299-3p to enhance SLC38A1 expression (Liu et al., 2022). SLC7A11 has been confirmed to be regulated by TP53. SLC38A1 and SLC7A11 belong to different family members of solute carrier (SLC) and participate in the regulation of ferroptosis. Whether TP53 and SLC38A1 have a regulatory relationship deserves further attention and research.

TP53 is reported to be involved in autophagy. DNA damage-regulated autophagy modulator and TP53-targeted autophagy-related genes, including unc-51-like autophagy activating kinase one and autophagy-related 7, are also essential for TP53-mediated apoptosis. Cytoplasmic TP53 inhibits AMP-dependent kinase, which, in turn, activates MTOR, leading to the inhibition of autophagy, whereas nuclear TP53 has the opposite effect. Mild stressors activate TP53, which, in turn, upregulates the level of basal autophagy without activation of MTOR-dependent induced autophagy. TP53/p53-FBXO22-TFEB controls basal autophagy, regulating hormesis (Suzuki et al., 2021). Increased levels of p53 acetylation suppress renal tubular epithelial cells (RTEC) autophagy after sepsis. Autophagy activation induced by p53 following deacetylation by Sirt1 reduces sepsis-associated acute kidney injury (SAKI) (Sun et al., 2021). UBE2T upregulates autophagy in NSCLC cells by activating the p53/AMPK/mTOR signaling pathway (Zhu et al., 2021).

CANX encodes a member of the calnexin family of molecular chaperones. Blocked mitosis in hypoxic cells may be due to the fact that after CANX knockout, the number of elongated mitochondria is increased, and the colocalization of autophagosomes and mitochondria is reduced (Wu et al., 2016). The endoplasmic reticulum (ER)-resident lectin chaperone Calnexin (CANX) and ER-phagy receptor FAM134B are required for autophagy-mediated quality control of endogenous procollagens (Forrester et al., 2019). VAMP3 is a member of the vesicle-associated membrane protein (VAMP)/synaptobrevin family. VAMP3 is required for the fusion between autophagosomes and multivesicular bodies (MVBs) in K562 cells (Fader et al., 2009). The STX6-VTI1B-VAMP3 complex facilitates xenophagy by regulating the fusion between recycling endosomes and autophagosomes (Nozawa et al., 2017). TP53 is not only an autophagy-related gene but also an important and potent ferroptosis-related gene. Revealing its role in IDD autophagy and regulatory axis of TP53-CANX/TP53-VAMP3 may inspire new ideas for the research on the regulatory network of IDD autophagy.

GNAI3 belongs to the G-alpha family. GNAI3 reduces autophagy flux and inhibits the autophagic degradation of hepatitis B virus antigen by enhancing the insulin-induced AKT-MTOR pathway (Wang et al., 2022). The activator of G-protein signaling 3 (AGS3), which is directly bound to light chain 3 (LC3), recruits Gαi3 to the LC3-positive membrane upon starvation and enhances autophagy by inhibiting G protein. Gα-interacting vesicle-associated protein (GIV) destroys the Gαi3-AGS three complex under the stimulation of growth factors, releases Gαi3 from the LC3-positive membrane, and inhibits autophagy by activating G protein (Garcia-Marcos et al., 2011). At present, there is no research involving GNAI3 and its encoded Gαi3 in the field of IDD. Elucidation of the role of GNAI3 in IDD may contribute to a further understanding of autophagy in IDD.

The present study had some limitations. First, the data used were downloaded from public databases, and it was not possible to evaluate whether some of those data contained errors. Second, only IDD tissue samples were analyzed, but a comprehensive analysis of venous blood samples and IDD tissue may be more helpful with respect to providing a general understanding of the potential mechanisms involved in IDD, which would be an improvement for future research. Lastly, a large amount of evidence is still needed to support our hypothesis. In subsequent studies, we will use various experiments, such as western blotting, immunohistochemistry assays, and dual luciferase reporter gene assays, to verify our conclusions and further clarify the mechanisms of IDD autophagy and ferroptosis.

In conclusion, the purpose of this study was to investigate the molecular mechanisms involved in IDD progression *via* comprehensive bioinformatics analysis to complement the understanding of autophagy and ferroptosis in IDD. This study might lead to the discovery of new IDD biomarkers, and more research is required to validate our results.

## Data Availability

The datasets presented in this study can be found in online repositories. The names of the repository/repositories and accession number(s) can be found in the article/Supplementary Material.
