# Corrigendum: A microarray data analysis investigating the pathogenesis and potential biomarkers of autophagy and ferroptosis in intervertebral disc degeneration

**DOI:** 10.3389/fgene.2023.1236744

**Published:** 2023-09-05

**Authors:** Wenhao Kuang, Cong Jiang, Cheng Yu, Jinwei Hu, Yang Duan, Zhong Chen

**Affiliations:** Department of Spinal Surgery, Zhujiang Hospital, Southern Medical University, Guangzhou, China

**Keywords:** autophagy, DEGs, ferroptosis, hub genes, integrated bioinformatics, intervertebral disc degeneration

In the published article, there was an error in Figure 7 and its **caption**. The incorrect figure was used for publication and the inequalities in the caption were missing a 0:

“Expression levels of hub genes in normal NPCs and degenerated NPCs. *p < .05; **p < .01; ***p < .001; ns, not significant.”

The correct Figure 7 and its **caption** appear below.

**FIGURE 7:**
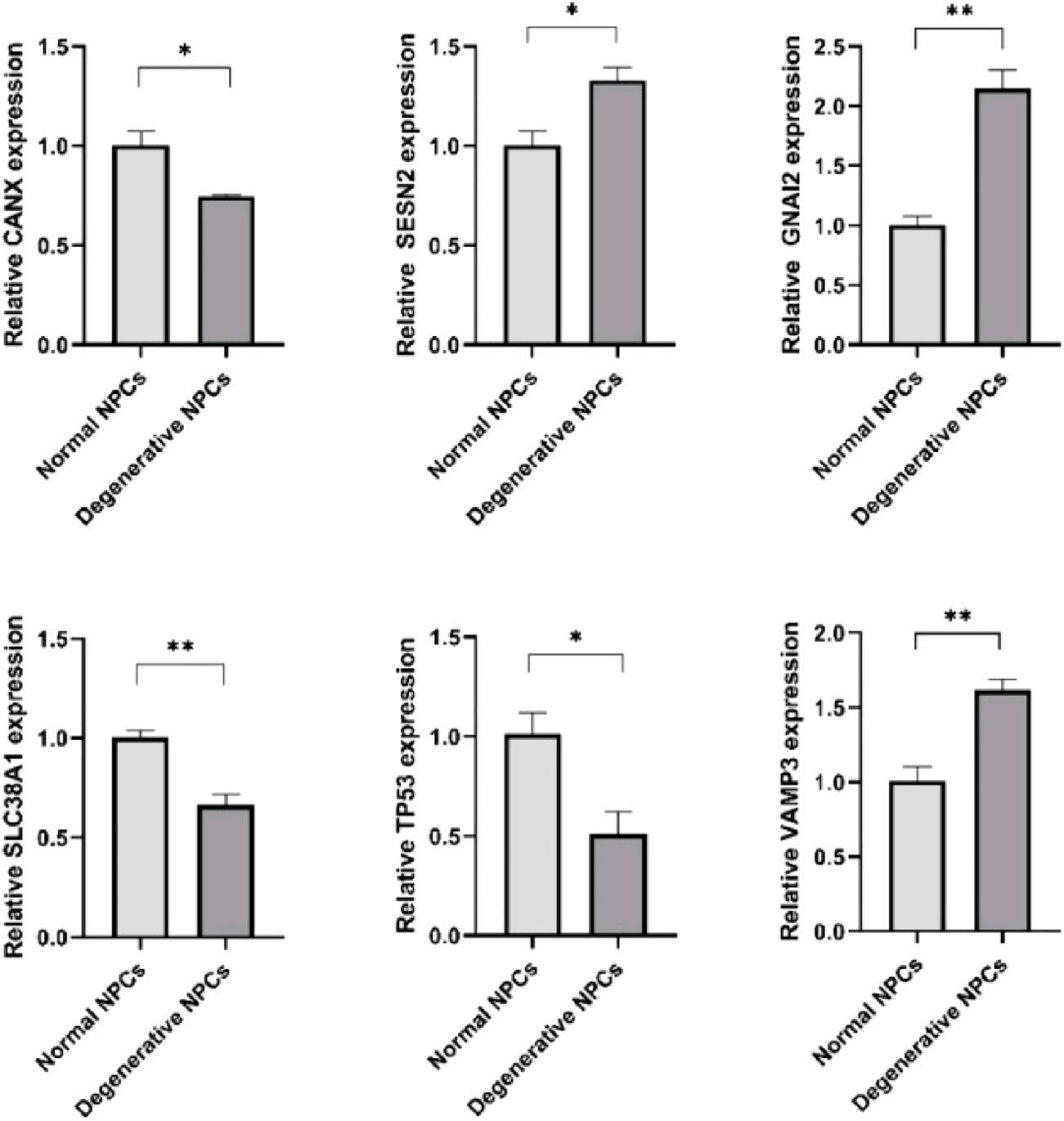
Expression levels of hub genes in normal NPCs and degenerated NPCs. *p < 0.05; **p < 0.01; ***p < 0.001; ns, not significant.

In the published article, there was an error in **Statistical analysis**, paragraph one. The word “one” was used instead of “independent”:

“Gene expression levels of clinical samples were compared using the one-sample t-test.”

The corrected sentence appears below:

“Gene expression levels of clinical samples were compared using the independent sample t-test.”

In the published article, there was an error in **Materials and methods**, *Isolation and culture of nucleus pulposus cells*. The word “first” was used instead of “fifth”:

“The cell culture medium was replaced on the first day and every two or three days thereafter.”

The corrected sentence appears below:

“The cell culture medium was replaced on the fifth day and every two or three days thereafter.”

The authors apologize for these errors and state that this does not change the scientific conclusions of the article in any way. The original article has been updated.

