# Supplementary material for: A microarray data analysis investigating the pathogenesis and potential biomarkers of autophagy and ferroptosis in intervertebral disc degeneration
Source: Front Genet. 2023 Jan 4;13:1090467. doi: 10.3389/fgene.2022.1090467 (PMC9846041; doi:10.3389/fgene.2022.1090467)
Supplement: Supplementary file 1 [file Table1.DOCX]

| Table1 89 hub genes | | | | | | | |
| --- | --- | --- | --- | --- | --- | --- | --- |
| ATP6AP1 | CANX | CCND2 | CDC42 | COL1A1 | EEF1A1 | IGFBP5 | LASP1 |
| PRELP | PTMA | SET | TP53 | EIF4H | MATR3 | ZC3H11A | CELF1 |
| BNC2 | TMEM214 | SLC38A1 | GNAI3 | MYO10 | PPIC | FOXP1 | FAM46A |
| THBS2 | RGS5 | HYPK | SERPING1 | FUS | GM2A | NDUFC2 | RPL12 |
| TOR1AIP1 | TNPO2 | ANXA4 | TRAM1 | CMTM6 | SETD3 | CAPZA1 | HLA-A |
| NAV1 | PRKAR2A | TMED2 | SULF2 | APLP2 | HMGN2 | DAZAP2 | ADAR |
| SGK1 | VAMP3 | SESN2 | GPX1 | ASPH | ZFP36L1 | MAT2A | PDGFRA |
| TMEM248 | COL1A2 | GNB1 | CRTAP | TMED10 | SCRG1 | ITGA11 | GPBP1 |
| ZCCHC24 | HLA-B | PSMB5 | RPL35 | DYNLT1 | CYP1B1 | PPT1 | SUMO2 |
| NFE2L1 | TRIM29 | VOPP1 | RPL27A | MSN | CLU | PGAM1 | DHX40 |
| FBXO28 | GRB2 | AMOTL2 | NRP2 | UGDH | TPM3 | TGOLN2 | SSR1 |
| UBLCP1 |  |  |  |  |  |  |  |
