# Supplementary material for: A microarray data analysis investigating the pathogenesis and potential biomarkers of autophagy and ferroptosis in intervertebral disc degeneration
Source: Front Genet. 2023 Jan 4;13:1090467. doi: 10.3389/fgene.2022.1090467 (PMC9846041; doi:10.3389/fgene.2022.1090467)
Supplement: Supplementary file 2 [file Table2.DOCX]

Table2 The GO and KEGG enrichment analyses of hub genes in IDD

| ONTOLOGY | ID | Description | Counts | p.adjust |
| --- | --- | --- | --- | --- |
| BP | GO:0070208 | protein heterotrimerization | 3 | 0.04370571 |
|  | GO:0002474 | antigen processing and presentation of peptide antigen via MHC class I | 5 | 0.04370571 |
|  | GO:0007596 | blood coagulation | 8 | 0.04370571 |
|  | GO:0006613 | cotranslational protein targeting to membrane | 5 | 0.04370571 |
|  | GO:0002478 | antigen processing and presentation of exogenous peptide antigen | 6 | 0.04370571 |
|  | GO:0007599 | hemostasis | 8 | 0.04370571 |
|  | GO:0050817 | coagulation | 8 | 0.04370571 |
|  | GO:0019884 | antigen processing and presentation of exogenous antigen | 6 | 0.04388518 |
|  | GO:0048002 | antigen processing and presentation of peptide antigen | 6 | 0.04788005 |
|  | GO:0071230 | cellular response to amino acid stimulus | 4 | 0.04894962 |
|  | GO:0051291 | protein heterooligomerization | 5 | 0.04894962 |
|  | GO:0072593 | reactive oxygen species metabolic process | 7 | 0.04894962 |
|  | GO:0034248 | regulation of cellular amide metabolic process | 9 | 0.04894962 |
| CC | GO:0042588 | zymogen granule | 3 | 0.0078053 |
|  | GO:0005788 | endoplasmic reticulum lumen | 8 | 0.00806168 |
|  | GO:0030662 | coated vesicle membrane | 6 | 0.01064114 |
|  | GO:0012507 | ER to Golgi transport vesicle membrane | 4 | 0.01064114 |
|  | GO:0071556 | integral component of lumenal side of endoplasmic reticulum membrane | 3 | 0.01246273 |
|  | GO:0098553 | lumenal side of endoplasmic reticulum membrane | 3 | 0.01246273 |
|  | GO:0055038 | recycling endosome membrane | 4 | 0.01704253 |
|  | GO:0031091 | platelet alpha granule | 4 | 0.02127679 |
|  | GO:0030134 | COPII-coated ER to Golgi transport vesicle | 4 | 0.02127679 |
|  | GO:0005583 | fibrillar collagen trimer | 2 | 0.02127679 |
|  | GO:0042589 | zymogen granule membrane | 2 | 0.02127679 |
|  | GO:0098643 | banded collagen fibril | 2 | 0.02127679 |
|  | GO:0055037 | recycling endosome | 5 | 0.02127679 |
|  | GO:0005798 | Golgi-associated vesicle | 5 | 0.02127679 |
|  | GO:0030660 | Golgi-associated vesicle membrane | 4 | 0.0257672 |
|  | GO:0030133 | transport vesicle | 7 | 0.0257672 |
|  | GO:0030135 | coated vesicle | 6 | 0.0257672 |
|  | GO:0031588 | nucleotide-activated protein kinase complex | 2 | 0.0257672 |
|  | GO:0062023 | collagen-containing extracellular matrix | 7 | 0.02799188 |
|  | GO:0030658 | transport vesicle membrane | 5 | 0.02942768 |
|  | GO:0005938 | cell cortex | 6 | 0.02942768 |
|  | GO:0022625 | cytosolic large ribosomal subunit | 3 | 0.03300919 |
|  | GO:0034774 | secretory granule lumen | 6 | 0.03300919 |
|  | GO:0098644 | complex of collagen trimers | 2 | 0.03581022 |
|  | GO:0060205 | cytoplasmic vesicle lumen | 6 | 0.03803232 |
|  | GO:0031983 | vesicle lumen | 6 | 0.03803232 |
|  | GO:0033116 | endoplasmic reticulum-Golgi intermediate compartment membrane | 3 | 0.03803232 |
|  | GO:0030176 | integral component of endoplasmic reticulum membrane | 4 | 0.04144671 |
|  | GO:0030670 | phagocytic vesicle membrane | 3 | 0.04288623 |
|  | GO:0031227 | intrinsic component of endoplasmic reticulum membrane | 4 | 0.04649678 |
|  | GO:0042611 | MHC protein complex | 2 | 0.0478303 |
| MF | GO:0048407 | platelet-derived growth factor binding | 3 | 0.00533266 |
| KEGG | hsa05165 | Human papillomavirus infection | 11 | 0.00073907 |
|  | hsa04510 | Focal adhesion | 8 | 0.00250796 |
|  | hsa04151 | PI3K-Akt signaling pathway | 10 | 0.00294227 |
|  | hsa04145 | Phagosome | 6 | 0.01445851 |
|  | hsa05163 | Human cytomegalovirus infection | 7 | 0.0152956 |
|  | hsa04926 | Relaxin signaling pathway | 5 | 0.03419516 |
|  | hsa05203 | Viral carcinogenesis | 6 | 0.03483872 |
|  | hsa05205 | Proteoglycans in cancer | 6 | 0.03483872 |
|  | hsa04512 | ECM-receptor interaction | 4 | 0.04114587 |
|  | hsa04218 | Cellular senescence | 5 | 0.04747178 |
