# Supplementary material for: A microarray data analysis investigating the pathogenesis and potential biomarkers of autophagy and ferroptosis in intervertebral disc degeneration
Source: Front Genet. 2023 Jan 4;13:1090467. doi: 10.3389/fgene.2022.1090467 (PMC9846041; doi:10.3389/fgene.2022.1090467)
Supplement: Supplementary file 3 [file Table3.DOCX]

Table 3 Primer sequences

| Gene | Forward primer | Reverse primer |
| --- | --- | --- |
| CANX | TACCAGCCTCAGCCTCCCAAAG | GCCAAGATCACGCCACTACACTC |
| GNAI3 | TTGGCACTGGCATCCTTGTCTTATC | TGCTCCTCCCTCAGTCTTCTCATTC |
| SESN2 | GAGGCAGGAGAATCGCTTGAACC | TTGAGACGGAGTATCGCTCTTGTTG |
| SLC38A1 | GGCACCACAGGGAAGTTCGTAATC | ACGATGAAGAGGTAGCTCAGCATTG |
| TP53 | AGGACAAGAAGCGGTGGAGGAG | TGTTGTTGGGCAGTGCTAGGAAAG |
| VAMP3 | GAGGCAGGAGAATGGCATGAACC | ACGGAGACTTGCTCTGTCACCTAG |
| GADPH | GCACCGTCAAGGCTGAGAAC | TGGTGAAGACGCCAGTGGA |
